# Diagnostic Screening of Bovine Mastitis Using MALDI-TOF MS Direct-Spotting of Milk and Machine Learning

**DOI:** 10.3390/vetsci10020101

**Published:** 2023-01-31

**Authors:** Jonathan Thompson, Savana L. Everhart Nunn, Sumon Sarkar, Beth Clayton

**Affiliations:** 1 School of Veterinary Medicine, Texas Tech University, 7671 Evans Dr., Amarillo, TX 79106, USA; 2 Dairy Herd Improvement Association, 301 23rd St., 117B, Canyon, TX 79015, USA

**Keywords:** diagnosis, mastitis, MALDI-TOF, somatic cell count, intramammary infection, machine learning, random forest, gradient-boosted trees, deep learning, decision tree, dairy, bovine

## Abstract

**Simple Summary:**

Monitoring health status and disease outbreak among food animal herds is vitally important to global food safety. Affected animals will experience production losses, and in uncurable cases, operations will need to be modified or animals culled. Producers and veterinary personnel have consistent interest in new diagnostic tools to provide rapid, accurate, and simple testing strategies which do not undermine financial viability of the operation. This manuscript describes a new approach for diagnosis of mastitis in dairy animals. The new method involves first analyzing raw milk from animals by matrix-assisted laser desorption/ionization mass spectrometry and collecting mass spectra. Then, peaks from the mass spectra are imported into a machine learning model, and this software application discovers non-obvious patterns present in the data which coincide with the mastitis condition. Finally, a separate set of milk samples is analyzed (scoring set) to evaluate the diagnostic accuracy of the new model. Results suggest that certain machine learning models offer value to the producer for diagnosis of subclinical mastitis in dairy cows. More generally, the manuscript outlines use of the machine learning approach for diagnoses of animal disease, and we prophesize that this strategy may be applicable to a wide array of animal health concerns.

**Abstract:**

Novel strategies for diagnostic screening of animal and herd health are crucial to contain disease outbreaks, maintain animal health, and maximize production efficiency. Mastitis is an inflammation of the mammary gland in dairy cows, often resulting from infection from a microorganism. Mastitis outbreaks result in loss of production, degradation of milk quality, and the need to isolate and treat affected animals. In this work, we evaluate MALDI-TOF mass spectrometry as a diagnostic for the culture-less screening of mastitis state from raw milk samples collected from regional dairies. Since sample preparation requires only minutes per sample using microvolumes of reagents and no cell culture, the technique is promising for rapid sample turnaround and low-cost diagnosis. Machine learning algorithms have been used to detect patterns embedded within MALDI-TOF spectra using a training set of 226 raw milk samples. A separate scoring set of 100 raw milk samples has been used to assess the specificity (*spc*) and sensitivity (*sens*) of the approach. Of machine learning models tested, the gradient-boosted tree model gave global optimal results, with the Youden index of *J* = 0.7, *sens* = 0.89, and *spc* = 0.81 achieved for the given set of conditions. Random forest models also performed well, achieving *J* > 0.63, with *sens* = 0.83 and *spc* = 0.81. Naïve Bayes, generalized linear, fast large-margin, and deep learning models failed to produce diagnostic results that were as favorable. We conclude that MALDI-TOF MS combined with machine learning is an alternative diagnostic tool for detection of high somatic cell count (SCC) and subclinical mastitis in dairy herds.

## 1. Introduction

Mastitis is an inflammation of the mammary gland, usually caused by infection by a microorganism. All female mammals are prone to the condition; however, in the dairy industry, mastitis has vast and far-reaching economic impacts on operations, since milk appearance, quality, composition, and yield can all be affected. Clinical mastitis is diagnosed when the body’s inflammatory response is extreme enough to change the appearance of the milk, mammary gland, or cow. Raw milk will appear brown, black, or red in color and/or exhibit extensive clotting making it unfit for sale and consumption. In subclinical cases, changes in milk composition or appearance may not be easily detected, but the milk may be high in somatic cell count (SCC), indicating presence of an infection. It is generally accepted that an SCC of ≥200,000 cells/mL in milk indicates high likelihood of infection, whether clinical signs are apparent or not [1]. Milk yield can be reduced by 5–10%—even at this early onset stage [2]. This may cost a dairy approx. 3 USD per day in production losses alone from a single animal. Additional expenses for the treatment of affected animals would be expected. Indeed, estimates of the economic costs of mastitis cases in dairy cows range from USD 179 to USD 444 per animal [3,4]. Thus, early detection of subclinical mastitis cases is of diagnostic interest to allow for rapid intervention to prevent further development of the condition in individual animals and outbreaks within herds.

Current practices for diagnosis of clinical mastitis include basic techniques such as udder and teat palpation and visualization of blood, clots, or flakes in the milk. More advanced techniques are required for subclinical mastitis detection, including somatic cell evaluation, plate-culture methods, pH, electrical conductivity, enzymatic activity, molecular diagnostic tools, and biosensors [5]. One of the most common diagnostic methods for cow-side subclinical mastitis detection is the California mastitis test (CMT). The CMT reagent lyses leukocytes, causing the release of DNA that forms a gel. The amount of gelation corresponds with the number of leukocytes within the milk sample. There are five results for the CMT: negative (approximately 0–200,000 SCC), trace (150,000–500,000 SCC), +1 (400,000–1 million SCC), +2 (800,000–5 million SCC), and +3 (>5 million SCC) [6].

At present, the concentration of somatic cell counts per milliliter of milk is considered the preferred metric for subclinical mastitis diagnosis since it is indicative of immune response to infection. Frequently, the dichotomous decision threshold of 200,000 cells/mL is used [7]. In this work, subclinical mastitis will be defined according to the following SCC thresholds: subclinical mastitis: >200,000 cells/mL; healthy cows: <200,000 cells/mL. However, there is no particularly high performing diagnostic test available to veterinarians, as common tests do not offer the combination of sensitivity/specificity as well as access, timeliness, and cost-effectiveness. For instance, Malek dos Reis et al. determined the sensitivity and specificity of various SCC count thresholds for diagnosis of mastitis in cows and found sensitivity values ranging from 0.37 to 0.74 when using SCC count thresholds from 400,000 → 100,000 cells/mL, respectively [8]. Corresponding specificity values were 0.81–0.50 over the same threshold range. In a separate work, Sargeant et al. considered the use of both SCC counts and the in-field CMT for diagnosis of mastitis state [9]. These authors found sensitivity values ranging from 0.65 to 0.98 when cell count thresholds of 500,000 → 100,000 cells/mL were used as diagnostic thresholds. However, specificity was low, ranging from 0.46 to 0.04 for the same SCC range. If the CMT diagnostic was considered, sensitivity ranged from 0.046 to 0.66, with specificity corresponding to 0.97–0.6. Kandeel et al. also evaluated the CMT within a well-controlled veterinary hospital setting [10]. These authors report sensitivity ranging between 0.05 and 0.45 and specificity of 0.98–0.56 for cows not presenting clinical mastitis symptoms. For cows with clinical mastitis, sensitivity ranged from 0.36 to 0.84, with specificity ranging from 0.82 to 0.38. Here, the ranges for the variables vary based upon the cut-point threshold used for the CMT test. These authors conclude that the CMT does not provide sufficient test sensitivity to identify quarters and dairy cows with an IMI on admission to a veterinary hospital. Front-line diagnostic methods relied upon by producers for detection of subclinical mastitis are available but offer less than optimal sensitivity and specificity.

Considerable efforts have also gone into understanding the chemical changes which occur within mastitic milk samples. Select reports have correlated small molecules such as lactose with mastitis [11]. Other authors have reported that levels of immune-related proteins such as cathelicidins, IGK, CD59, and lactadherin, as well as protease inhibitors, change significantly upon onset of mastitis [12]. Smolenski et al. report on a comprehensive analysis of proteins in milk by 2D electrophoresis and liquid chromatography–mass spectrometry (LC–MS) that uncovered the presence of over 2700 proteins, with approx. 15 attributed to host defense mechanisms [13]. Taken together, these works suggest that the chemical composition of milk changes when mastitis occurs, and that, therefore, such changes may effectively be used as a diagnostic tool for sensing outbreaks of mastitis.

Matrix-assisted laser desorption/ionization time-of-flight mass spectrometry (MALDI-TOF MS) is now a well-established tool within the research community for intact protein mass analysis and microorganism identification [14,15]. The analytical benefits of MALDI-TOF are rapid and facile sample preparation, and analysis of a wide variety of biomolecules in molecularly intact form, with limited matrix interference. Thus, MALDI-TOF MS is a promising platform for a variety of diagnostic tests if biomarkers are known or can be identified. While some biomarkers have been identified (see previous paragraph), the change in chemical composition of milk after the onset of mastitis is still not fully characterized. Thus, additional analytes may provide valuable diagnostic information.

A significant challenge for the analyst is identifying analytes to use for dichotomous diagnostics. One promising approach to assist in the identification of biomarkers and improved diagnoses is use of machine learning technologies [16,17,18,19,20,21,22]. In machine learning, patterns are detected within data sets using statistical inferences embedded within the data to facilitate dichotomous decision making. In one such work, Ebrahimi et al. evaluated various machine learning models (deep learning (DL), naïve Bayes, generalized linear model, logistic regression, decision tree, gradient-boosted tree (GBT) and random forest) using data from 364,249 milking instances in which milk volume, lactose concentration, electrical conductivity (EC), protein concentration, peak flow, and milking time were measured for each sample [17]. Using these parameters, the authors achieved a sensitivity of >93% for diagnosis of the mastitis condition. However, diagnostic specificity was more of a struggle, as only 1.2–40% was achieved for the various models.

Given the rich chemical information provided by a MALDI TOF spectrum and the pattern-recognition ability of machine learning, the two techniques are a natural fit for analytical diagnostics. An analyst would certainly struggle to diagnose mastitis from a MALDI mass spectrum without prior knowledge of biomarkers indicating the condition. However, if a set of sample spectra is used to train a machine learning algorithm, patterns may be uncovered which lead to diagnostic accuracy.

In this study, the objective was to evaluate the performance of machine-learning-based diagnostics utilizing a MALDI TOF MS data stream for the detection of high SCC, and, consequently, subclinical mastitis state in dairy cows. Several machine learning models were explored for performance, including decision trees, random forest, gradient-boosted trees, naïve Bayes, generalized linear model, fast large-margin model, and deep learning. Model performance was evaluated by receiver operating characteristic (ROC) curve analysis and computation of model sensitivity (*sens*), specificity (*spc*), and the Youden index (*J*). The approach tested should be applicable to periodic screening of herds for mastitis state.

## 2. Materials and Methods

**Figure 1:**
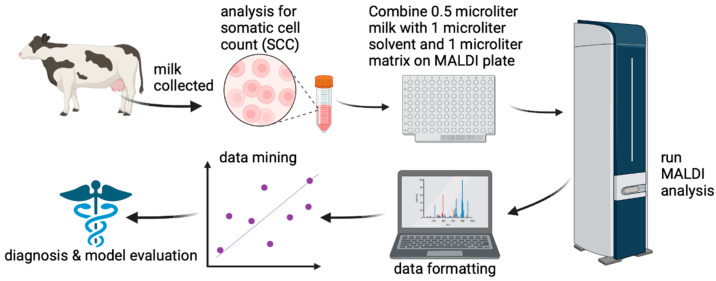
Overview of project work flow.

An overview of the project workflow is depicted in Figure 1 below. First raw milk was collected into 50 mL plastic vials. These samples were submitted for somatic cell counting and chemical analysis at a partner laboratory. Then, samples were provided to our laboratory for testing as samples of opportunity. Next, milk was mixed with MALDI matrix and spotted on a plate, prior to mass spectral analysis. The resultant data were formatted prior to data mining and model evaluation.

### 2.1. Samples and Sample Preparation

#### 2.1.1. Raw Milk Samples

Samples of raw milk were obtained from the Texas Dairy Herd Improvement Association (DHIA) laboratory in Canyon, TX. These samples were originally collected at dairies throughout Texas and submitted to the DHIA laboratory for analysis. After completing their analysis, DHIA provided aliquots of the residual samples to our laboratory as samples of opportunity for our research. The samples were obtained from individual cows as a composite after the udder was completely milked out. Samples were collected as the cows came into the milking parlor, which was randomly. However, some samples were selected with knowledge that the cows were within a hospital pen (i.e., affected by clinical mastitis) in an effort to acquire sufficient numbers of positive samples for analysis. The DHIA laboratory conducted analysis of milk SCC, fat, protein, and lactose using a Milkoscan FT+ spectrometer (Foss) and provided these results in a spreadsheet file for use in this project. DHIA uses a preservative (bronopol), which was added to all samples at 0.02%. The breed of animal and location of dairies providing quarter samples were not revealed to protect confidentiality. For the direct spotting method, N = 226 individual samples were used to create the training data set for machine learning. Of these samples, N = 129 presented with SCC < 200,000 and were deemed negative for mastitis (thus the diagnostic threshold was SCC > 200 k cells/mL). The additional N = 97 had SCC > 200,000 and were deemed mastitis positive. Either the ‘*negative*’ or the ‘*positive*’ identifier was added to the working data spreadsheet for each sample.

An additional and separate set of N = 100 composite milk samples was obtained from DHIA for use as the scoring data set. Of these samples, SCC results provided by DHIA indicated mastitis in N = 53 of the composite samples, with the remaining 47 negative. These samples were spotted and analyzed in an identical procedure to that used for the training data set, but analysis was conducted on a different day. In addition, the scoring-data-set composite milk samples were obtained from a different local dairy.

#### 2.1.2. Sample Preparation and Spotting

Milk samples were applied to the MALDI plate via direct spotting. Briefly, 0.5 μL of raw milk was deposited upon the MALDI target, and then 1 μL of solvent containing a mixture of 1:1:1 acetonitrile, ethanol, and water with 3% trifluoroacetic acid (TFA) was added. Finally, 1 μL of a saturated solution of α-cyano-4-hydroxycinnamic acid (CHCA) matrix was mixed and the mixture allowed to dry prior to analysis.

### 2.2. MALDI-TOF-MS Analysis

#### MALDI-TOF Mass Spectra Acquisition

Data were acquired using a Shimadzu Axima Performance MALDI-TOF mass spectrometer. Laser power was set at 74 (arbitrary units) and 50 Hz repetition rate. An automated rastering pattern was used over 1000 profiles collected, with 2 pulses per profile. A mass range of 500–16,000 *m*/*z* in the linear TOF positive ion mode was employed. MALDI TOF calibration was performed prior to analysis using a peptide mixture of known masses between *m*/*z* = 757–3658 Da (LaserBioLabs, Valbonne, France) and horse heart cytochrome C for *m*/*z* up to 12,351 Da. Peak centroid masses observed were within 0.5–1 Da of the accepted mass for all calibrants.

### 2.3. Data Mining

#### 2.3.1. Data Formatting

After acquisition of a MALDI spectrum, the Axima Performance software was used to identify/pick peaks of centroid masses and record data to a comma delimited text file. This step is crucial to provide an automated and reproducible method to identify peaks and quantify signal strength. Next, the peak list files were formatted using a program written in-house (LabView, National Instruments, Austin, TX, USA). The data mining software requires consistent labels for *m*/*z* to find patterns within the data stream. Small differences in *m*/*z* from run-to-run would be considered different labels by the data miner if not corrected. Thus, this computer program begins at the user-defined lower limit of *m*/*z* = 500 Da and queries the peak list file if a peak is present within ±1 Da. If found, the integer *m*/*z* and peak intensity are written to a new text file. If no peak is present, the integer *m*/*z* and a zero are passed to the file. The code then iterates to the original *m*/*z*, adds 2 Da to the mass, and repeats the process until the upper limit of *m*/*z* is reached (here upper limit is *m*/*z* = 16,000 Da). This results in a text file with peak data from 500 to 16,000 Da, with resolution of 2 Da. This is a necessary step to smooth the data effectively and present it to the data mining software with the *m*/*z* value essentially as a categorical entry. The mining software uses *m*/*z* only as a label for categorical data, such as gender, state of residence, etc., when data mining is used for finding patterns in consumer data. After formatting all files, mass spectral data were compiled in a spreadsheet and transposed.

#### 2.3.2. Data Mining Models and Runs

All data mining was accomplished within RapidMiner Studio software (Version 9.10, RapidMiner GmbH). Models used included decision tree, random forest, gradient- boosted trees, naïve Bayes, generalized linear model, fast large-margin, and deep learning. For all models, mastitis state (negative or positive) was identified as the ‘label’ within the software, indicating that this was the value to be predicted for the scoring samples in a dichotomous manner. Each model has its own variables which can be adjusted by the user in the software. Within the Results section below, we describe efforts to vary these variables in more detail. Upon each model run, the training data set was used to construct the model and the scoring data set used to evaluate. Each data mining run used the identical experimental data. Data mining results were saved to a spreadsheet file indicating mastitis state according to the SCC results used as the reference, and the data mining model prediction. Then, diagnostic sensitivity (*sens*) and specificity (*spc*), as well as the Youden index, were computed for the 100 scoring-set milk samples, using the typical approach [23].

## 3. Results

### 3.1. Machine Learning Models and Model Evaluation

#### 3.1.1. Decision Trees

**Figure 2:**
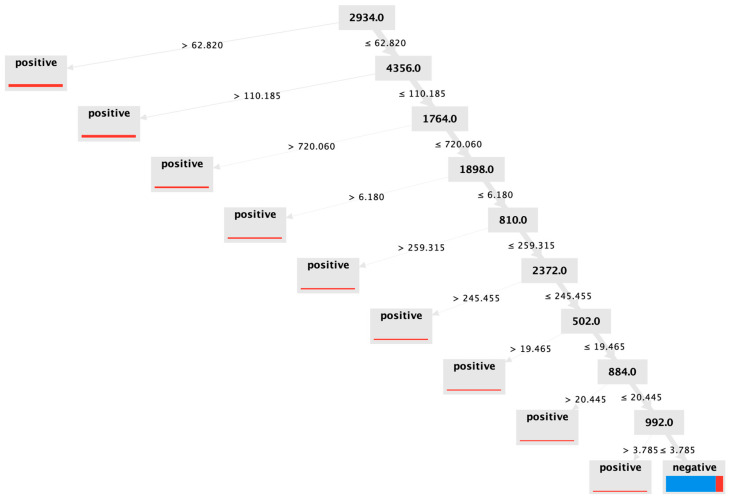
Decision tree model generated by RapidMiner for the learning/training set. The numbers within grey boxes represent *m*/*z* values from MALDI spectra—thus they are useful guides to identify potential biomarkers indicative of mastitis. This decision tree represents the model, trained from 226 samples of known SCC. In the bottom right corner, 129/148 samples were correctly sorted as negative. Conversely, 78/97 positive samples were correctly sorted during model building.

Decision trees represent a logical, intuitive, and highly visual means to report machine learning data. The tree is simply a set of logical binary decisions based upon mass-to-charge (*m*/*z*) values and intensity signals observed at each *m*/*z* within the MALDI mass spectrum. An example decision tree obtained during experiments is shown below in Figure 2.

This tree begins by considering the *m*/*z* = 2934. If the signal observed was ≥62.82, the sample was always positive for mastitis in the training set. Even if the signal were below this threshold, the sample may still be positive. The remaining logical operators throughout the tree classify the training set samples accordingly. In this training set tree, only 19 samples deemed positive for mastitis using the SCC metric were grouped with the 129 negative results. When this decision tree is used to sort the separate 100 milk scoring/evaluation samples, we obtain a result of 69% correct diagnosis, with *sens* = 0.679 and *spc* = 0.702. The decision tree model performance is reasonably good (though not the global optimum obtained). However, the most significant advantage of using decision trees is the ease with which analysts can identify potential biomarkers. Since the values within the grey boxes correspond to *m*/*z* values for peaks observed in the mass spectrum, and the model identified these markers as key features for sorting data into groups for ‘*negative*’ and ‘*positive*’, the substances generating these peaks appear to be relevant to physiology.

**Figure 3:**
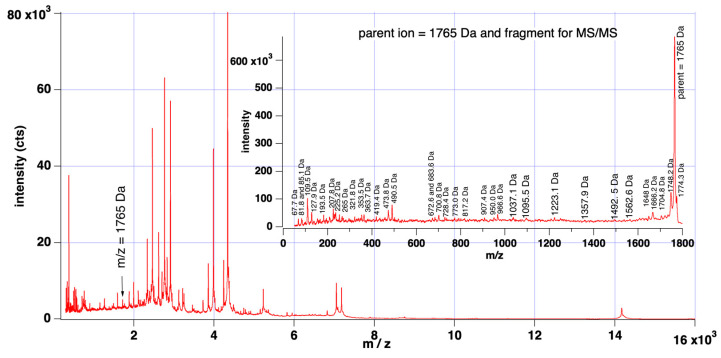
MALDI mass spectrum of milk from cows affected by mastitis. Inset is MALDI TOF/TOF spectrum of parent *m*/*z* = 1765 Da after fragmentation by collision-induced dissociation (CID). The peak at 1765 Da is indicated as a key ion in the decision tree.

Interestingly, the peptide identified at 1898 Da is a fragment of serum amyloid A protein (see supplementary data), which is known to be secreted during periods of inflammation and has previously been found upregulated within milk affected by mastitis [24]. MALDI TOF/TOF experiments were also conducted to elucidate molecular structures of key peaks identified in the decision tree, as shown in Figure 3. However, fragment peaks have not been conclusively matched to peptides. The peak observed in the MALDI spectrum at 1765 Da (labelled as 1764 in Figure 2 above) presents as being a most crucial spectral element, as 116/128 negative results could be differentiated by this peak.

In RapidMiner, decision trees have user-adjustable parameters including splitting criterion, tree maximal depth, confidence, and minimal gain for splitting. For criteria, the accuracy, information gain, and gain ratio has been explored. Maximal tree depth was 2–20, confidence was 0.01–0.5, and minimum gain ranged between 0.001 and 0.15. *Sens* ranged from 0.62 to 0.943, with *spc* corresponding to 0.82–0.064 over the range tested. The Youden index (J) is the maximal distance between a receiver operating characteristic (ROC) curve value and the diagonal line which describes diagnostic futility [25]. The Youden index can be computed by:

*J* = {(*sens* + *spc*) − 1} (1)

The Youden index is often described as the optimal criterion value for balancing *sens* and *spc* for dichotomous diagnostic decisions, though limitations exist on this statement depending on the consequences of positive/negative results. For the decision tree analysis, *J* = 0–0.509, indicating a wide range of diagnostic performance. The best performing model (*J* = 0.509) was the ‘accuracy’ criterion, with >10 tree depth. For this set of conditions, *sens* = 0.849, with *spc* = 0.659. The intuitive and visual nature of decision trees makes them appealing; however, alternative machine learning approaches yielded higher performance (see below).

#### 3.1.2. Random Forest

**Figure 4:**
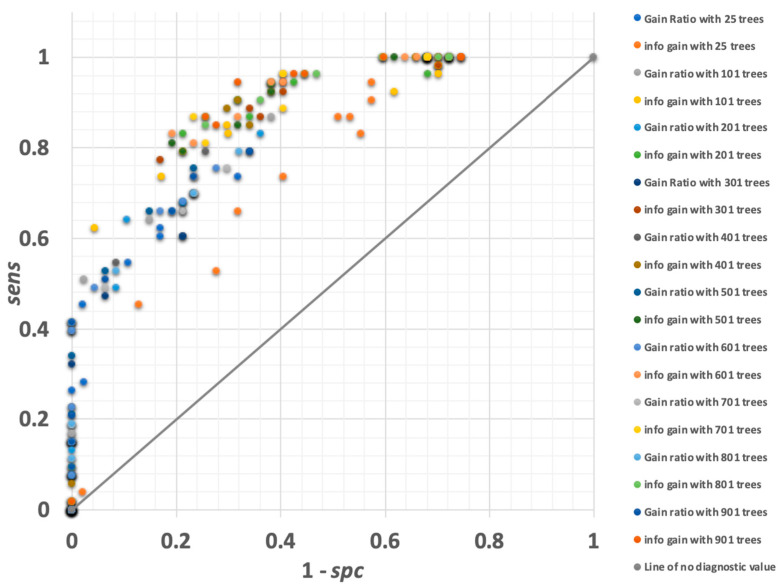
ROC curve for all random forest models explored. Models differ by adjusting parameters within RapidMiner. ‘Gain ratio’ and ‘info gain’ refer to criteria on which attributes will be selected for splitting within RapidMiner software. The optimal random forest model performed well, achieving *sens* = 0.83, *spc* = 0.81, and 82% of the scoring milk samples assigned to the correct diagnostic category.

Random forest models create *N* decision trees based on the training data set and then apply these trees to the scoring data set. A decision is reached for each sample by simple voting, or a confidence-weighted voting process. Within RapidMiner, the random forest operator has several user adjustable parameters, including number of trees, decision criterion, maximal depth of trees, and voting strategy. Using the same training and scoring data sets, we have varied these parameters in a systematic study of how they affect model performance. Figure 4 reports a receiver operating characteristic curve (ROC) for the random forest models explored. As observed, chosen parameters for the random forest model affected performance dramatically. Poorest performance was observed when using the gain-ratio model—particularly when large number of decision trees were employed. To an extent, this may be counterintuitive as large number of decision trees may be perceived to offer a modelling advantage. Optimal performance was observed when *J* = 0.6385 was achieved, for a random forest model which was based on the information gain criterion, confidence polling, maximal tree depth of five branches, and N = 601 individual decision trees. For this model, *sens* = 0.83, *spc* = 0.81, and 82% of the scoring milk samples were assigned to the correct category.

#### 3.1.3. Naïve Bayes Model

In RapidMiner, the naïve Bayes operator has no user adjustable parameters. Thus, only one model-build and scoring evaluation was necessary. The naïve Bayes model successfully predicted 64% of the scoring-set data correctly for mastitis state with *sens* = 0.868 and *spc* = 0.383. As other models outperformed it, this model was not pursued further.

#### 3.1.4. Generalized Linear Model

The generalized linear model was used with default parameters of AUTO for family and solver, with regularization, standardize, and add intercept boxes checked. This model predicted 68% of measurements correctly, with *sens* = 0.9245 and *spc* = 0.4043. If regularization was not used but other parameters remained the same, 66% prediction accuracy was achieved, with *sens* = 0.925 but *spc* = 0.36. When the generalized linear model was employed with regularization, lambda search, and early stopping, the model predicted the mastitis state of 69% of samples correctly, with *sens* = 0.868 and *spc* = 0.49. This performance was the highest observed for the generalized linear model, but with a Youden index of *J* = 0.285–0.370, this model option was not investigated further, since its performance was far lower than that of other models, and no further improvements in performance could be achieved.

#### 3.1.5. Fast Large-Margin Model

The fast large-margin model was also evaluated. For this set of experiments, the Ls SVM Dual, L2 SVM Primal, and L2 Logistic Regression solvers were used. Penalty cost (C) was varied from 1 to 1000, and the termination criterion epsilon was varied between 0.1 and 1000. Numerous conditions were used, resulting in prediction accuracy of 53–71%. Values for *sens* ranged from 0.58 to 1.0; however, corresponding *spc* values were 0.85–0. The Youden index was *J* = 0–0.436, so this option was also not investigated further, since its performance was considerably lower than that of other models, and no further improvements in performance could be demonstrated.

#### 3.1.6. Gradient-Boosted Trees

**Figure 5:**
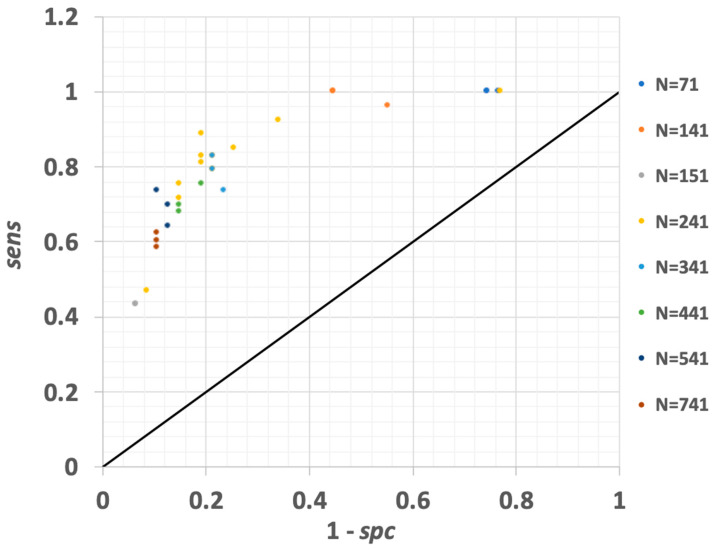
ROC curve for all gradient-boosted tree models explored. Models differ by adjusting parameters within RapidMiner. Here, N corresponds to number of trees. See text for remaining parameters adjusted. The gradient-boosted tree model performed the best of all models tested, achieving *sens* = 0.89, with *spc* = 0.81 and 85% of the scoring milk samples assigned to the correct diagnostic category for optimal model conditions. A project-maximum Youden index was achieved for this model when *J* = 0.7.

The gradient-boosted tree model has many adjustable parameters including number of trees, maximal depth, minimum rows, number of bins, learning rate, sample rate, and distribution. During investigative trials, the number of trees was varied between 71 and 741, with maximal depth from 4 to 10, minimum rows 5–10, bins 10–30, learning rate 0.01–0.75, split improvement 0.00005–0.001, and sample rate 0.5. Figure 5 reports the ROC curve for the gradient-boosted model runs tested. The gradient-boosted model offers high performance, as a trial using 241 trees, depth of 4, 10 rows, 30 bins, 0.05 learning rate, and 0.0001 split improvement yielded the maximal performance of any model tested during our efforts. For this model build, *sens* = 0.89 was achieved, with *spc* = 0.81 and 85% diagnostic accuracy. The Youden index was *J* = 0.7 for this global optimum trial. As observed in Figure 5, a cluster of high-performing cases occurred when 241–341 trees were used in the model. Increasing the number of trees to N = 541 or 741 did not increase performance.

#### 3.1.7. Deep Learning Model

**Figure 6:**
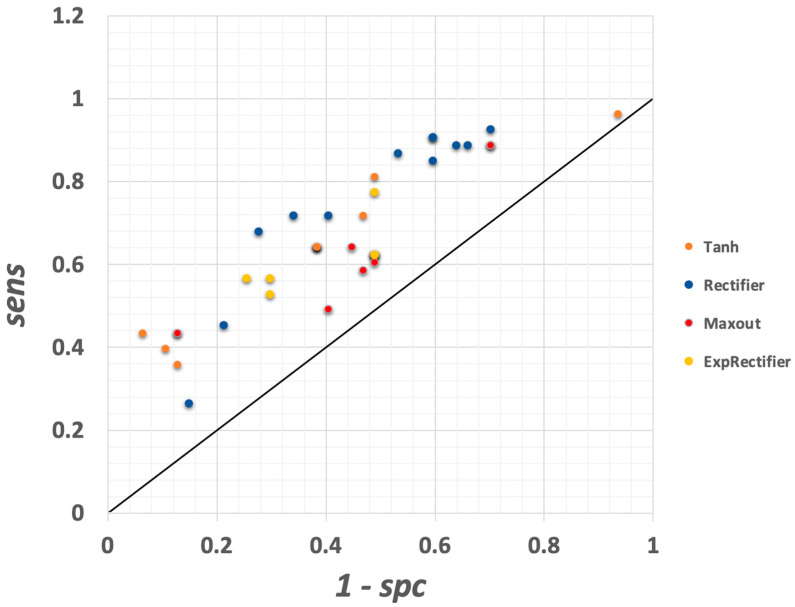
ROC curve for all deep learning models explored. Models differ by adjusting parameters within RapidMiner. See text for remaining parameters adjusted. Maximal performance for deep learning was *sens* = 0.68 and *spc* = 0.72, with *J* = 0.4.

The final model considered in this study was deep learning and an ROC curve depicting results is illustrated in Figure 6 below. This model features adjustable parameters for activation, reproducible model, epochs, epsilon (learning rate), rho, among several others. For exploration, the activation parameter was toggled between Tanh, Rectifier, Maxout, and ExpRectifier within RapidMiner. Epochs ranged between 10 and the max allowed (1.8e308). Eleven of the model runs used the ‘reproducible’ setting, while the remaining runs did not. It should be noted that replicate runs of the deep learning model with the same input data may result in slightly different output results. The deep learning model runs produced scoring-set results of between 54 and 70% diagnostic accuracy. Additional figures of merit included *sens* ranging from 0.26 to 0.96 and *spc* ranging from 0.06 to 0.94. The optimum model run was achieved when Rectifier was used with maximal allowed epochs. However, this model run resulted in only *sens* = 0.68 and *spc* = 0.72, with *J* = 0.4, a level of performance considerably lower than what was achieved for the random forest and gradient-boosted models. The Deep Learning model was not pursued further, since additional gains in performance could not be demonstrated.

## 4. Discussion

In this study, the performance of machine-learning-based diagnostics utilizing a MALDI-TOF MS data stream for the detection of subclinical mastitis, according to a predefined SCC threshold (subclinical mastitis: >200,000 cells/mL; healthy cows: <200,000 cells/mL) was assessed by receiver operating characteristic (ROC) curve analysis and computation of model sensitivity (*sens*), specificity (*spc*), and the Youden index (*J*). Results indicate that the random forest and gradient-boosted trees machine-learning models perform the best of all approaches tested.

Results suggest the working hypotheses that MALDI-TOF MS coupled with machine learning is a valuable tool for subclinical mastitis diagnosis. As observed in Table 1, metrics for *sens* and *spc* exceed values reported previously in the literature for the SCC diagnostic alone and the California mastitis test (CMT) test. Both the random forest and gradient-boosted models developed within the current study have matched or exceeded the diagnostic performance reported in Schepers et al. [26], a reference work which is largely responsible for establishing the use of SCC as a diagnostic for mastitis in dairy herds.

Since MALDI-TOF MS measurements can be automated and require minimal sample preparation, analytical turnaround times may be on the order of a few hours if an instrument is available. In addition, since only microvolumes of reagents are required, analysis can be inexpensive on a per sample basis—costing roughly 1 USD; however, this figure ignores the substantial cost to acquire MALDI TOF.

While this study strongly suggests that coupling MALDI TOF to machine learning is a promising diagnostic tool, the work is limited by several factors. First, samples used were collected from regional dairies, and it is not clear if the training set would be applicable to cows from other regions or climates in which husbandry practices and diet differ. It is also not clear whether teat infections caused by bacteria different from those common in West Texas would alter the MALDI TOF spectra so dramatically as to affect results. If training-set data cannot be extrapolated to a variety of locations, the need will exist to re-train the diagnostic algorithm, requiring significant investment of time and resources. In addition, while running samples using MALDI TOF is inexpensive, acquisition of the mass spectrometer is not. The device typically costs several hundred thousand US dollars, making the technique inaccessible to many dairies and veterinary professionals.

Future directions of work could focus on the use of decision trees to further investigate/identify novel biomarkers of mastitis. In addition, a more thorough economic cost/benefit analysis of using MALDI TOF MS for mastitis diagnoses could be carried out. Towards this latter goal, assessing the applicability of training data sets to samples from a variety of locations would begin to allow understanding of how generally applicable the technique truly is. While initial results are promising, follow-up replication and expansion of sample sets will produce further development of this field.

## 5. Conclusions

Matrix-assisted laser desorption/ionization time-of-flight mass spectrometry (MALDI-TOF) data have been coupled with machine learning algorithms to develop an analytical diagnostic for subclinical mastitis in dairy cows. As discussed above, the diagnostic outperforms alternate existing options for testing and presents an exciting alternative for future work. The approach described within this manuscript is, in principle, applicable to a wide variety of veterinary diagnostic tests, and it is expected that machine learning coupled with MALDI data streams will be applied to many diagnoses in future years. However, additional effort must be focused on creating widely applicable training data set libraries so that small differences in animal lifestyle do not confound successful implementation of the diagnostic.

## Figures and Tables

**Table 1:** Optimal *sens, spc*, and *J*_max_ observed for several machine learning models and comparison literature. Here, *J_max_* is maximal Youden index.

Model	*sens*	*spc*	*J*_max_
Decision tree	0.85	0.66	0.51
Random forest	0.83	0.81	0.64
Naïve Bayes	0.87	0.38	0.25
Gen. linear	0.87	0.49	0.37
Fast large-margin	0.58	0.85	0.43
Grad. boosted trees	0.89	0.81	0.70
Deep learning	0.68	0.72	0.40
Comparison case SCC * [10]	0.59	0.72	0.31
Comparison case CMT ** [12]	0.27	0.85	0.12
Comparison case SCC [26]	0.74	0.90	0.64

* Somatic cell count (SCC) threshold of 200k/mL; study optimum reported. ** California mastitis test (CMT), test threshold of +1, any infection.

## Data Availability

Spreadsheets containing data for the training set and scoring set are available online at the Texas Tech University ThinkTech web repository. Re-use of the data stream for other purposes beyond normal scholarly activity related to the current manuscript is allowed only with written permission of the authors.

## Supplementary Materials

The following supporting information can be downloaded at: https://www.mdpi.com/article/10.3390/vetsci10020101/s1, Supplementary Materials file which contains two Excel spread-sheets with original data. References [27,28] are cited in the supplementary materials.

## References

[1] Mastitis in Cattle—Reproductive System—Merck Veterinary Manual. https://www.merckvetmanual.com/reproductive-system/mastitis-in-large-animals/mastitis-in-cattle.

[2] Janzen J.J. (1970). Economic Losses Resulting from Mastitis. A Review. J. Dairy Sci..

[3] Liang D., Arnold L.M., Stowe C.J., Harmon R.J., Bewley J.M. (2017). Estimating US Dairy Clinical Disease Costs with a Stochastic Simulation Model. J. Dairy Sci..

[4] Rollin E., Dhuyvetter K.C., Overton M.W. (2015). The Cost of Clinical Mastitis in the First 30 Days of Lactation: An Economic Modeling Tool. Prev. Vet. Med..

[5] Martins S.A.M., Martins V.C., Cardoso F.A., Germano J., Rodrigues M., Duarte C., Bexiga R., Cardoso S., Freitas P.P. (2019). Biosensors for On-Farm Diagnosis of Mastitis. Front. Bioeng. Biotechnol..

[6] Peek S.F., Divers T.J. (2018). Rebhun’s Diseases of Dairy Cattle.

[7] Schukken Y.H., Wilson D.J., Welcome F., Garrison-Tikofsky L., Gonzalez R.N. (2003). Monitoring Udder Health and Milk Quality Using Somatic Cell Counts. Vet. Res..

[8] dos Reis C.B.M., Barreiro J.R., Moreno J.F.G., Porcionato M.A.F., Santos M.V. (2011). Evaluation of Somatic Cell Count Thresholds to Detect Subclinical Mastitis in Gyr Cows. J. Dairy Sci..

[9] Sargeant J.M., Leslie K.E., Shirley J.E., Pulkrabek B.J., Lim G.H. (2001). Sensitivity and Specificity of Somatic Cell Count and California Mastitis Test for Identifying Intramammary Infection in Early Lactation. J. Dairy Sci..

[10] Kandeel S.A., Morin D.E., Calloway C.D., Constable P.D. (2018). Association of California Mastitis Test Scores with Intramammary Infection Status in Lactating Dairy Cows Admitted to a Veterinary Teaching Hospital. J. Vet. Intern. Med..

[11] Antanaitis R., Juozaitienė V., Jonike V., Baumgartner W., Paulauskas A. (2021). Milk Lactose as a Biomarker of Subclinical Mastitis in Dairy Cows. Animals.

[12] Zhang L., Boeren S., van Hooijdonk A.C.M., Vervoort J.M., Hettinga K.A. (2015). A Proteomic Perspective on the Changes in Milk Proteins Due to High Somatic Cell Count. J. Dairy Sci..

[13] Smolenski G., Haines S., Kwan F.Y.S., Bond J., Farr V., Davis S.R., Stelwagen K., Wheeler T.T. (2007). Characterisation of Host Defence Proteins in Milk Using a Proteomic Approach. J. Proteome Res..

[14] Chen X.-F., Hou X., Xiao M., Zhang L., Cheng J.-W., Zhou M.-L., Huang J.-J., Zhang J.-J., Xu Y.-C., Hsueh P.-R. (2021). Matrix-Assisted Laser Desorption/Ionization Time of Flight Mass Spectrometry (MALDI-TOF MS) Analysis for the Identification of Pathogenic Microorganisms: A Review Matrix-Assisted Laser Desorption/Ionization Time of Flight Mass Spectrometry (MALDI-TOF MS) Analysis for the Identification of Pathogenic Microorganisms: A Review. Microorganisms.

[15] Li D., Yi J., Han G., Qiao L. (2022). MALDI-TOF Mass Spectrometry in Clinical Analysis and Research. ACS Meas. Sci. Au.

[16] Roscher R., Bohn B., Duarte M.F., Garcke J. (2020). Explainable Machine Learning for Scientific Insights and Discoveries. IEEE Access.

[17] Ebrahimi M., Mohammadi-Dehcheshmeh M., Ebrahimie E., Petrovski K.R. (2019). Comprehensive Analysis of Machine Learning Models for Prediction of Sub-Clinical Mastitis: Deep Learning and Gradient-Boosted Trees Outperform Other Models. Comput. Biol. Med..

[18] Vervier K., Mahé P., Veyrieras J.-B., Vert J.-P. (2015). Benchmark of Structured Machine Learning Methods for Microbial Identification from Mass-Spectrometry Data.

[19] Weis C.V., Jutzeler C.R., Borgwardt K. (2020). Machine Learning for Microbial Identification and Antimicrobial Susceptibility Testing on MALDI-TOF Mass Spectra: A Systematic Review. Clin. Microbiol. Infect..

[20] Esener N., Guerra A.M., Giebel K., Lea D., Green M.J., Bradley A.J., Dottorini T. (2021). Mass Spectrometry and Machine Learning for the Accurate Diagnosis of Benzylpenicillin and Multidrug Resistance of Staphylococcus Aureus in Bovine Mastitis. PLoS Comput. Biol..

[21] Tran N.K., Howard T., Walsh R., Pepper J., Loegering J., Phinney B., Salemi M.R., Rashidi H.H. (2021). Novel Application of Automated Machine Learning with MALDI-TOF-MS for Rapid High-Throughput Screening of COVID-19: A Proof of Concept. Sci. Rep..

[22] Piras C., Hale O.J., Reynolds C.K., Jones A.K., Taylor N., Morris M., Cramer R. (2022). LAP-MALDI MS Coupled with Machine Learning: An Ambient Mass Spectrometry Approach for High-Throughput Diagnostics. Chem. Sci..

[23] Stevenson M. (2008). An Introduction to Veterinary Epidemiology.

[24] Thomas F.C., Mullen W., Tassi R., Ramírez-Torres A., Mudaliar M., McNeilly T.N., Zadoks R.N., Burchmore R., David Eckersall P. (2016). Mastitomics, the Integrated Omics of Bovine Milk in an Experimental Model of Streptococcus Uberis Mastitis: 1. High Abundance Proteins, Acute Phase Proteins and Peptidomics. Mol. Biosyst..

[25] Youden W.J. (1950). Index for Rating Diagnostic Tests. Cancer.

[26] Schepers A.J., Lam T.J.G.M., Schukken Y.H., Wilmink J.B.M., Hanekamp W.J.A. (1997). Estimation of Variance Components for Somatic Cell Counts to Determine Thresholds for Uninfected Quarters. J. Dairy Sci..

[27] Mudaliar M., Tassi R., Thomas F.C., McNeilly T.N., Weidt S.K., McLaughlin M., Wilson D., Burchmore R., Herzyk P., Eckersall P.D. (2016). Mastitomics, the Integrated Omics of Bovine Milk in an Experimental Model of Streptococcus Uberis Mastitis: 2. Label-Free Relative Quantitative Proteomics. Mol. Biosyst..

[28] Yu W., Zhao S.-L. LC/MS/MS Analysis of Melamine in Liquid Milk and Milk Powder with Bond ElutPlexa PCX. https://www.agilent.com/chem.

