# Supplementary material for: Diagnostic Screening of Bovine Mastitis Using MALDI-TOF MS Direct-Spotting of Milk and Machine Learning
Source: Vet Sci. 2023 Jan 31;10(2):101. doi: 10.3390/vetsci10020101 (PMC9962131; doi:10.3390/vetsci10020101)
Supplement: Supplementary file 1 [file vetsci-10-00101-s001.zip › Supplementary Materials new.pdf]

Supplementary Materials

"Diagnostic Screening of bovine Mastitis using MALDI-TOF MS Direct-Spotting of Milk and Machine Learning."

Overview: One of the benefits of using a decision tree model is it identifies m/z values from the MALDI spectrum which differentiate between disease and non-diseased states. This allows the analyst to perform follow-up experiments to attempt identification of the key biomarkers. We have attempted this type of experiment using both MALDI TOF MS/MS (see Fig. 3 in manuscript) and LC-MS of mastitis milk. The LC-ESI-MS method is briefly described here. The analysis was able to definitively identify the fragment at m/z = 1898 Da as peptide SGKDPNHFRPAGLPDKY, a marker of inflammation in milk.

Method: The LC-MS instrumental method was adapted from the work of Mudaliar et al. [1] and sample preparation was adapted from Yu and Zhao [2], except that solid phase extraction was not performed.

Results: The LC and MS data collected is reported in the figure below. In this figure, the top trace reports the base peak ion chromatogram (e.g. all peaks in chromatogram). This displays the full chemical diversity of milk sample. The second trace (magenta) reports a selected ion chromatogram for the target peptide SGKDPNHFRPAGLPDKY which was observed to elute as a minor component of the milk mixture between 50-60 minutes. The third figure reports the mass spectrum collected at 52.5 minutes into the run which shows a peak at m/z = 475.49 Da corresponding to the $z = +4$ ion of the target peptide. The peaks highlighted in yellow are additional peaks corresponding to the peptide of differing isotopes.

References:

(1) Mudaliar, M.; Tassi, R.; Thomas, F. C.; McNeilly, T. N.; Weidt, S. K.; McLaughlin, M.; Wilson, D.; Burchmore, R.; Herzyk, P.; Eckersall, P. D.; Zadoks, R. N. Mastitomics, the Integrated Omics of Bovine Milk in an Experimental Model of Streptococcus Uberis Mastitis: 2. Label-Free Relative Quantitative Proteomics. *Mol Biosyst* **2016**, *12* (9), 2748–2761. https://doi.org/10.1039/C6MB00290K.

(2) Yu, W.; Zhao, S.-L. *LC/MS/MS Analysis of Melamine in Liquid Milk and Milk Powder with Bond ElutPlexa PCX*. www.agilent.com/chem.

C:\Users\Thermo\Desktop\data04.raw 1/2/2023 12:40:39 PM

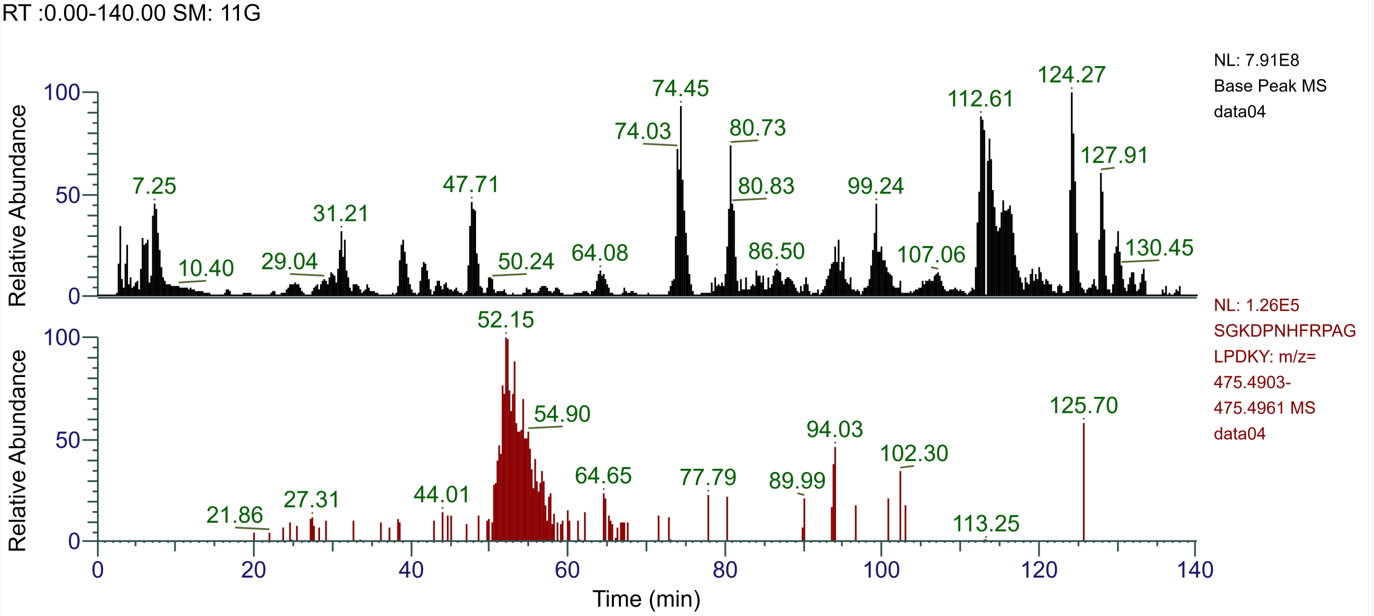

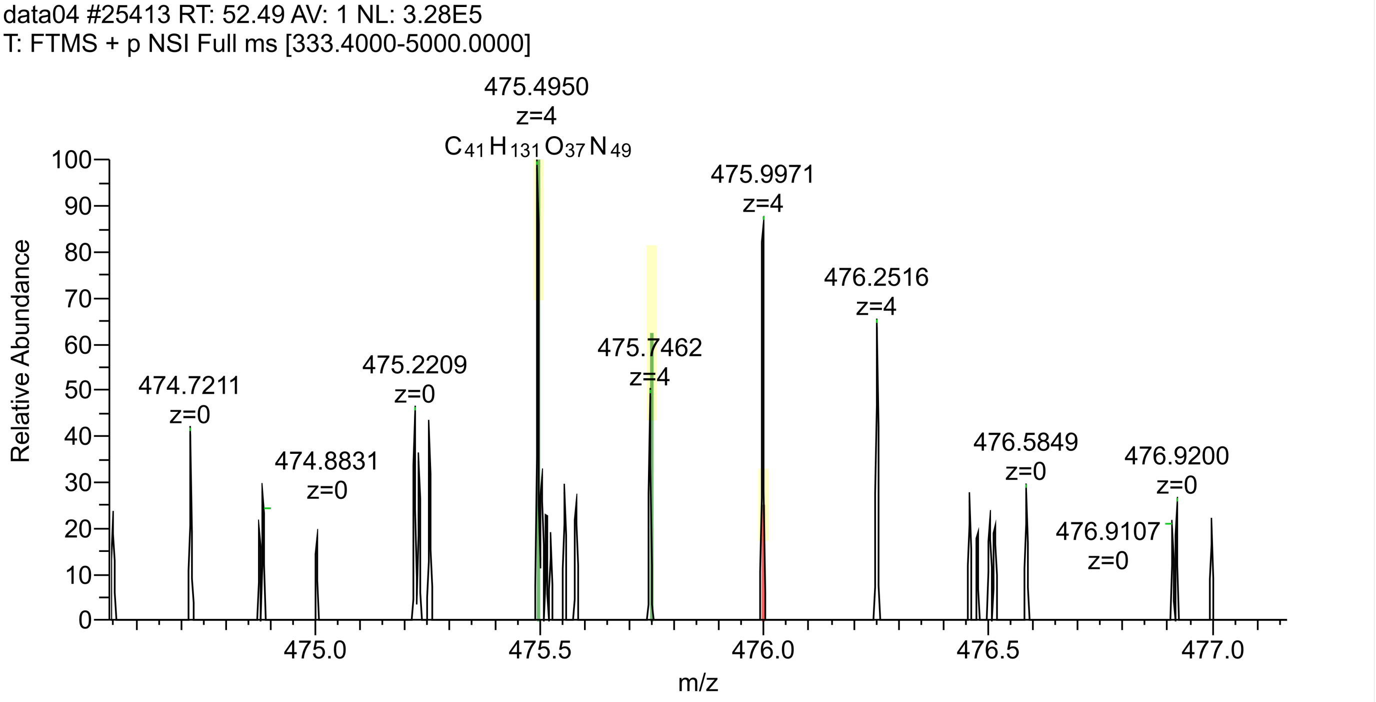

Peak Mass	Display F...	S Fit	RDB	Delta [pp...	Theo. ma...	Rank	Combine...	# Match...	# Missed...	MS Cov....	Pattern C...	MSMS M...
475.4950	$C_{41}H_{131}O_{37}N_{49}$	31.0361960802006	1.00	-2.82	475.49634	1	34.48	2	1	34.67	86.53	(Collection)
475.4950	$C_{47}H_{131}O_{36}N_{45}$	28.9960162339055	5.00	0.97	475.49454	2	34.37	2	1	34.67	85.57	(Collection)
475.4950	$C_{46}H_{135}O_{40}N_{41}$	28.1099531427392	0.00	1.67	475.49420	3	34.33	2	1	34.67	85.72	(Collection)
475.4950	$C_{50}H_{139}O_{42}N_{35}$	21.9050904074413	-1.00	0.26	475.49487	4	34	2	2	34.67	81.27	(Collection)
[illegible]	[illegible]	21.88429[illegible]	[illegible]	[illegible]	475.4970[illegible]	[illegible]	[illegible]	[illegible]	[illegible]	[illegible]	[illegible]	(Collecti[illegible]
